# A dynamical perspective: moving towards mechanism in single-cell transcriptomics

**DOI:** 10.1098/rstb.2023.0049

**Published:** 2024-04-22

**Authors:** Rory J. Maizels

**Affiliations:** ^1^ The Francis Crick Institute, 1 Midland Road, London NW1 1AT, UK; ^2^ University College London, London WC1E 6BT, UK

**Keywords:** single-cell genomics, machine learning, gene regulation, dynamical systems, causal inference

## Abstract

As the field of single-cell transcriptomics matures, research is shifting focus from phenomenological descriptions of cellular phenotypes to a mechanistic understanding of the gene regulation underneath. This perspective considers the value of capturing dynamical information at single-cell resolution for gaining mechanistic insight; reviews the available technologies for recording and inferring temporal information in single cells; and explores whether better dynamical resolution is sufficient to adequately capture the causal relationships driving complex biological systems.

This article is part of a discussion meeting issue ‘Causes and consequences of stochastic processes in development and disease’.

## 1. Introduction

Single-cell transcriptomics has provided wide-ranging insights into the varied processes driving development and disease. For example, revealing spatial and temporal codes of expression in the developing nervous system [1,2] illustrating the progression of cell states observed through gastrulation [3,4], and detailing complex phenotypes observed in diseases such as Alzheimer’s [5]. As single-cell technologies continue to expand, attention is shifting from seeking phenomenological descriptions of cellular phenotypes to the causal relationships in the regulatory mechanisms that drive these phenotypes [6].

Single-cell methods are well suited for such investigations: assaying the entire transcriptome at once reduces the risk of failing to observe important factors, resolving gene expression in individual cells can allow subtle behaviours to be detected, and by achieving these feats with thousands to millions of data points per experiment, single-cell approaches promise to properly grasp the complexity of biological systems.

Thus, it is no surprise that modelling gene regulation from single-cell data is a highly active area of research. However, such efforts have had mixed successes: for example, methods for inference of gene regulatory networks from single-cell data often perform little better than random guesswork [7,8]. Recently, several methods have been developed for modelling gene regulatory networks from joint single-cell assays of RNA and chromatin accessibility (reviewed elsewhere [9]), for example facilitating the identification of transcription factors driving developmental lineages [10]. However, while these models provide means to develop and explore hypotheses regarding key regulators, they fall short of providing a comprehensive or robust explanation of the logic of gene regulation in the systems studied.

An obstacle for constructing such models from single-cell data is the destructive, ‘snapshot’ nature of sequencing, which means that temporal dynamics are not captured at single-cell resolution. Analyses often rely on pseudotemporal ordering [11,12], which collapses the data into a time-series averaged across the population level. This article suggests that a dynamical perspective can be valuable for capturing the inherent temporal structure of causal relationships, considers the experimental and computational methods that can measure and infer temporal dynamics in single-cell data, and explores how these methods could integrate with other genomics technologies for analysis of gene regulation. Finally, we are left to ask whether these many technologies for measuring and inferring regulatory dynamics are sufficient, or whether we may need new ways to define the underlying causal relationships themselves.

## 2. Dynamics and mechanism

The term ‘mechanism’ has seen varied use through the history of science [13]. In the context of cellular and developmental biology, mechanism can describe the causal relationships between genes, or indeed the causal relationship between a set of interacting genes and the cellular phenotype they control or regulate. A causal mechanism in this context could consist of an explanation of how a particular set of genes affect a particular cellular process. For this explanation to be *mechanistic*, it would need to be to some degree both predictive and intuitive. To be predictive, it should be able to show how perturbations such as genetic knockouts would affect the system’s outputs. To be intuitive, it should provide an explanation, from the perspective of the system’s component parts, of the chain of events that lead such perturbations to have this effect.

A fundamental notion common to all causal relationships is temporal ordering: for *X* to cause *Y*, it must precede *Y*. As such, capturing temporal dynamics with sufficient resolution is necessary to discern whether *X* causes *Y*, or the reverse, or whether both are caused by the third factor *Z*. More concretely, there are particular notions describing the relationship between two factors that we can expect to find when that relationship is causal (discussed in more detail in [14]) such as the proportionality and specificity of the mapping from the causative factor to the effect, and the stability of the interaction across a range of different conditions. These notions are often considered in the context of interventionalist studies (e.g. examining the stability, proportionality, and specificity of effect for genetic knockouts), but causal relationships between genes may also be evident through stability of interactions in the face of stochastic and heterogenous behaviours of individual cells [15].

The challenge of distinguishing causal from correlative relationships becomes more critical as the systems being studied become more complex: gene regulatory networks are often cross-repressive, with many forms of feedback between genes. In these contexts, causality is time-dependent and cannot be adequately captured with simple atemporal counterfactuals (that is, ‘If *X* then *Y*’) [14]. Thus, a well-defined picture of dynamics is required not only to resolve the relationship between *X* and *Y*, but also the history and context of this relationship, which can determine how it manifests.

With conventional single-cell RNA sequencing technologies, temporal dynamics can be captured through pseudo-temporal ordering, where a time series is constructed by ordering cells from a define start to a define endpoint. The methods for doing so can differ considerably; from approaches that describe the minimum distance traversal across the graph that describes the data [16], to Bayesian approaches that model the data as a Gaussian process ([17]; for a review of some approaches, see [18]). The resultant temporal information is not truly at ‘single-cell’ level: it is always indirectly inferred from information aggregated across the wider population. Many different dynamic regimes at the cellular level can produce the same observed distributions at the population level [11], meaning that in the context of questions of gene regulation, subtle but important notions—such as stability and proportionality of interactions—may not be discernible from population level dynamics.

One could imagine a hypothetical gold standard: real-time measurements of transcriptomes over multiple time points, in individual cells. With this, one could record the relationship between genes through time and across the varying contexts of different cells, capturing structured correlations that would be masked in population-level data. For example, if gene *X* is induced at variable times across cells, but in the face of this variation gene *Y* is consistently induced shortly afterwards, this would provide more robust evidence of a relationship between *X* and *Y* than would be possible from simply observing *Y* follows *X* in the population average. By moving to single-cell resolution of time, one can capture nuanced notions of causality that would otherwise be blurred out.

While there have been some early efforts to sample transcriptomes from live cells without destruction (for example LIVE-seq [19]), these methods remain labour intensive and low-throughput. As such, the longitudinal measurements of this imagined gold standard are not yet possible with the cellular throughput or genomic breadth that single-cell sequencing provides. However, there are methods that can encode temporal information into sequencing data and computational methods that can infer underlying dynamics, taking us towards being able to model and simulate this gold standard of dynamical resolution.

## 3. Recording time in individual cells

RNA sequencing is generally a destructive assay. To capture temporal information, it can be encoded into the material that will eventually be collected and sequenced.

For example, one could record information about the age of the messenger RNA (mRNA) molecules that are sequenced. By partitioning detected molecules into time windows (for example, ‘new’ and ‘old’), one can get a measure of how gene expression is changing. In other words, such information would provide information that can be used to infer a cell’s gene expression ‘velocity.’

A widely used approach for this, RNA velocity [20], uses the inherent temporal ordering of unspliced pre-mRNA and spliced mRNA in sequencing data. By constructing a per-gene reaction model that compares the observed ratio of unspliced and spliced reads to an inferred expected ratio for a given cell and gene, this approach can approximate changing rates of mRNA synthesis. A higher ratio of unspliced to spliced mRNA than expected implies gene expression is increasing, while a lower ratio than expected implies gene expression is decreasing.

The power of this approach is its wide applicability to any dataset where spliced and unspliced reads are both detected—which is, surprisingly, most single-cell transcriptomics datasets. However, there are many conceptual caveats to this approach: many genes are not spliced, while many others undergo alternative splicing, and many more display dynamically regulated splicing behaviour. More generally, most single-cell RNA-seq methods capture mRNA by targeting poly-A tails, which should be absent in unspliced pre-mRNA. Hence, it may be that intronic reads are captured through unintentional binding of primers to intronic regions. The full extent of the effects of these various sources of technical and biological confounding factors is not clear, but it has been shown that unspliced mRNA detection suffers from gene length bias [21] consistent with unintentional intronic priming, and that between 20 and 40% of genes across tissues in mouse and human lack significant unspliced information [22], suggesting that splicing data may not provide a robust measurement of time across genes.

An alternative approach is to use an experimentally incorporated RNA label to distinguish new from pre-existing RNA. For example, 4-thiouridine (4sU) is a cell permeant uridine analogue that incorporates into nascently transcribed RNA. Through chemical treatment with iodoacetamide or trifluoroethylamine, this label can be converted to an analogue of cytosine. As such, transcripts produced after the addition of 4sU can be identified by a characteristic signature of U-to-C mutations.

**Figure 1.**
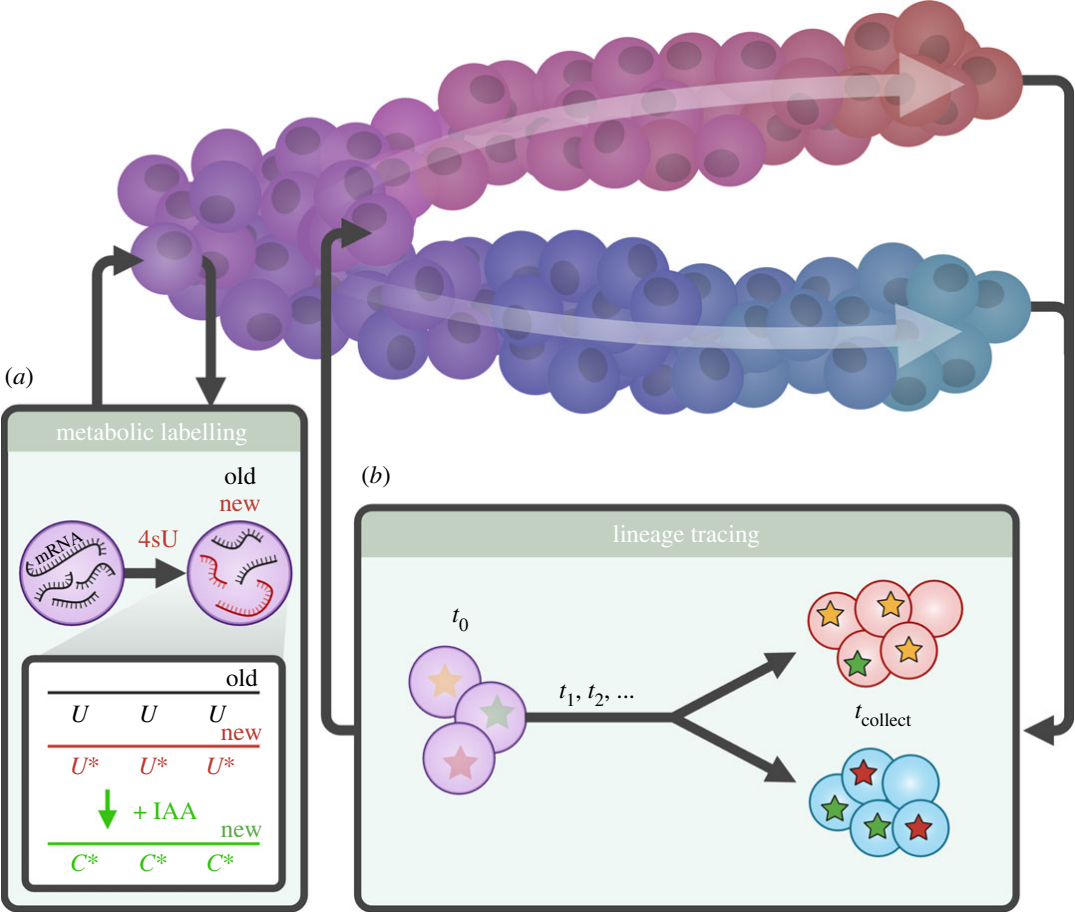
Capturing dynamics at single-cell resolution. (*a*) Molecular measurements of dynamics record temporal information regarding the molecules of mRNA detected within each individual cell. For example, metabolic labelling distinguishes ‘new’ from ‘old’ transcripts based on the incorporation of 4-thiouridine (4sU), which can be chemically converted into an analogue of cytosine that is detected in sequencing. IAA, iodoacetamide. (*b*) Cellular measurements of dynamics capture the temporal relationships between cells in an experiment. For example, lineage tracing involves incorporating an inheritable signature at an earlier timepoint (*t*_0_) and detecting these signatures in sequencing at a later timepoint, meaning clonal relationship between cells can be detected.

This approach, termed metabolic labelling (figure 1*a*), was first done in an RNA-seq protocol with SLAM-seq [23], before single-cell methods were developed (scSLAM-seq [24] and NASC-seq [25]). Since these approaches, there have been a number of methods developed that can facilitate single-cell metabolic labelling in thousands of cells per experiment: sci-FATE [26], scNT-seq [27], scEU-seq [28] (which uses ethynyl uridine instead of 4sU), well-TEMP-seq [29], SLAM-Drop-seq [30] and most recently sci-FATE2 [22]. Metabolic labelling experiments can now be performed in tens of thousands of cells per experiment, with overall data quality comparable to conventional single-cell RNA sequencing. With the combinatorial indexing approaches, sci-FATE and sci-FATE2, it is possible to introduce additional rounds of indexing to facilitate the collection of millions of cells per experiment.

Metabolic labelling can be thought to provide a *molecular* measurement of time, providing information about the temporal relationship between molecules of mRNA detected within each cell. A complementary approach would be a *cellular* measurement of time, that provides temporal information regarding the different cells collected in an experiment. One such approach is lineage tracing, which describes the clonal relationships between cells (figure 1*b*). In lineage tracing, each cell receives a unique, inheritable signature at one time point, and this signature is detected in progeny at a later timepoint: sequenced cells that share a signature have descended from the same ancestor at the initial timepoint. This describes temporal dynamics by revealing the range of fates reachable by the latter timepoint from a single cellular identity at the initial timepoint. By constructing this lineage marker as a barcode that can be sequenced, this assay can be carried out at the same time as single-cell RNA-seq, providing a definitive link between gene expression and cell fate. The exact method of introducing these signatures can vary considerably: they can be barcode sequences introduced through viral infection [11,31] or integration [32] into cells, they may be signatures of unique recombinations of transgenic constructs [33] or signatures of scarring induced by Cas9-induced double-strand breaks [34,35]; barcodes or signatures may be introduced only once, introduced cumulatively to provide a longitudinal map of cell lineages, or may in fact be naturally present in mitochondrial DNA ([36]; for more details see [37–39])—but in any case, lineage tracing provides a measure of fate commitment and clonal relationships that is orthogonal to the dynamics inferred by approaches such as RNA velocity and metabolic labelling. The timescales studied are considerably longer, and the relationships captured are different: cellular behaviour, rather than the molecular dynamics of the genes controlling this behaviour. As such, lineage tracing does not directly provide information about how genes may be interacting to regulate cellular behaviour, but can provide vital information to ensure that inferred dynamics are consistent with longer-term cellular dynamics [40,41].

Recent developments in the field of single-cell genomics show promise in expanding the available tools for both molecular and cellular measurements of time in single-cell datasets. For instance, RNA timestamping [42] employs the activity of adenosine deaminases acting on RNA (ADAR) to introduce A-to-G edits into RNA sequences sequentially over time. This method moves the molecular recording of time beyond the binary of ‘new’ and ‘old’, offering a continuous distribution of edits that can be used to approximate the age of individual molecules. For cellular recordings, DNA Typewriter [43] is a technique where barcoded information can be systematically incorporated into DNA in a temporally ordered manner. This method allows for the construction of a ‘tape’ of molecular recordings of as many as 20 sequential events, facilitating the recording of cellular histories beyond the scope of just mitotic lineages.

As our ability to encode temporal information into sequencing data grows, we are increasingly able to overcome the ‘snapshot’ limitation of sequencing technology. However, it is not enough to simply encode this information: the dynamics of the system must also be inferred.

## 4. Inferring single-cell dynamics

Molecular measurements of mRNA age, such as metabolic labelling, provide parallel measurement of nascent and prior expression. Using this data to model the synthesis and degradation rates for genes, one can estimate the time derivative of gene expression, or velocity, in a given cell, providing a better view of the temporal dynamics of gene expression.

The majority of such models are designed to work with splicing data. Many have been developed: firstly, velocyto [20], which frames velocity estimation as a linear regression of unspliced and spliced reads, and subsequently several methods built to address particular modelling caveats, such as steady-state assumptions [44] or modelling timescales [45,46]. More recently, a number of studies have applied machine learning, in particular deep generative modelling, to the problem of velocity inference from splicing data [19,47–50]. The motivation for such an approach is that while single-cell datasets may have thousands of dimensions, there exist in the data useful patterns that can be represented in a more tractable lower-dimensional space. Deep generative modelling (specifically, variational autoencoders [51]) employ the power and flexibility of neural networks to learn these hidden (or ‘latent’) representations of the data. In doing so, they can overcome the difficulties of working with high dimensional, sparse, noisy data. Deep generative modelling methods have seen success in other single-cell approaches [52] such as multi-modal integration [53] and data correction [54], and have now been applied to the task of improving dynamical inference with RNA velocity.

However, such approaches are still constrained by the underlying quality of splicing data and the modelling assumptions required to make velocity inference from splicing dynamics a well-posed task, and a recent comparison of RNA velocity inference tools found that many performed poorly when assessed against a ground truth of known biological dynamics [22].

Methods that use metabolic labelling data have expanded the scope of downstream analysis. For example, dynamo [55] learns a vector field in a low dimensional embedding by performing vector-valued regression on velocities that have been projected into the embedding. With this vector field, one can estimate dynamical properties of the system such as curvature and acceleration, and the most likely path between any two points according to the vector field can be calculated.

One area of particular potential is the application of neural differential equations [56] to model dynamics beyond the single timestep of instantaneous velocity. LatentVelo [48] and DeepVelo [19] both learn neural ordinary differential equations from splicing data, developing methods to learn long-term trajectories: LatentVelo assumes a single initial condition with a latent ‘regulatory state’, while DeepVelo limits prediction errors by re-mapping trajectories to nearby cells every few timesteps. Recent work, VelvetSDE [22], has extended to modelling neural stochastic differential equations [57,58] from metabolic labelling data to provide simulations of single-cell trajectories that recapitulate the distributions of the underlying data and allow prediction of state-fate maps.

As time-resolved transcriptomics modelling continue to develop, these methods move towards opening up new forms of analysis of gene regulatory mechanisms. In particular, the inference of gene expression velocity creates natural connections to tools from statistical mechanics and dynamical systems theory. These tools can provide understanding of complex processes from fluid dynamics to protein folding to the evolution of ecosystems [59]. Applied to cellular processes, they provide frameworks to reconstruct the regimes that link complex genotypic patterns to the phenotypic outcomes of developmental decisions or disease prognosis. By providing robust and theoretically principled tools for understanding complex and dynamic systems, statistical mechanics and dynamical systems theory can provide intuition and insight where they may be otherwise hard to find.

For example, the sequence of cell state transitions observed along a developmental trajectory can be modelled as an energy potential landscape [59–61], providing a geometrical description that can provide information on aspects such as the role of stochasticity and the potential for reversibility. In such frameworks, biological concepts can be recast from phenotypic descriptions into mathematic quantities: cell types are captured as basins of attraction, developmental lineages as minimum action paths. With more data-driven approaches for learning these mathematical quantities, it will be possible to explore the effects of perturbations through the predicted effects on the structure of inferred potential landscapes.

Combining single-cell genomics’ ability for comprehensive phenotypic profiling with the dynamic descriptions from statistical mechanics will create a particularly compelling framework for tackling the complexity of biological processes. With conventional single-cell RNA sequencing, the integration of these disciplines has been restricted to simple cases, such as binary fate decisions [62] and tristable networks ([63]; for a more comprehensive review, see [59]). Improving the quality of temporal information in data can only strengthen the connection between these fields, facilitating dynamical models with fewer user-inputted constraints, and allowing more nuanced behaviours to be accurately captured. However, there remain challenges to overcome in order to achieve this goal.

## 5. Dynamical challenges

For time-resolved sequencing methods to provide meaningful insight into the logic of gene regulation, a number of challenges will need to be overcome.

A simple but central challenge will be the continued improvement in the quality and affordability of single cell assays. Single-cell datasets suffer from high levels of noise, low sensitivity, and issues of ‘dropout’ for lowly expressed genes, all resulting from the fact that only a small portion of a cell’s transcriptome is ever captured for sequencing. While there may be practical and biophysical limits to the amount of information that can be captured in each cell, the upper limit for the number of cells captured per experiment appears more flexible, with recent sequencing methods developing a capacity of millions of cells per experiment [64,65]. Indeed, there is evidence that beyond a particular threshold, the benefits of greater sequencing depth per cell diminish relative to the benefit of sequencing more cells [66]. However, sequencing millions of cells remains time consuming and expensive, meaning that more straightforward and affordable methods will be needed if sequencing in the order of millions of cells is to be a realistic solution to the statistical challenges posed by dropout and noise in single-cell data.

More work is also required to address the noise and stochasticity inherent to biological systems themselves. While stochastic models of gene expression have been employed to address biophysical noise [44,67], biological variability must also be taken into account: cell behaviour is not necessarily deterministic, and accurately capturing the noise and uncertainty inherent in biological dynamics will be required for robust modelling of cellular behaviour.

The integration of metabolic labelling with lineage tracing could help in this respect: labelling provides a molecular measurement of short-term dynamics, while lineage tracing records what different states can be reached from a single state at an earlier timepoint. In other words, labelling can be used to model how cells are ‘moving’ through gene expression space, while lineage tracing can provide a measure of the variation, or uncertainty, in cellular trajectories. The two methods are complementary, integrating bottom-up and top-down perspectives for dynamical inference across different timescales and providing a unified view of instantaneous and long-term gene expression dynamics.

Another major challenge arises from the fact that RNA profiling provides an incomplete picture of gene regulation. For example, the action of a transcription factor is dependent not only on its own expression but also on multiple additional layers of regulation, including the accessibility of target sites in the genome. This particular confounding factor can be addressed through joint profiling of RNA and chromatin accessibility through multiomic RNA + ATAC sequencing. Indeed, the use of multiomics for studying gene regulatory networks is an active area of research, with many methods available (reviewed elsewhere [9]).

With information on chromatin accessibility, one can predict what potential targets of a transcription factor may or may not be acted upon in a particular context [68]. Such approaches aim to capture a more mechanistic view of gene regulation, recording a transcription factor’s expression and possible targets within the same cells. However, the use of chromatin accessibility data for this aim is not without challenges: open chromatin regions must be matched to particular transcription factors using binding motifs that can be flexible and degenerate [69]; and these chromatin regions must then be assigned as a *cis*-regulatory element for genes within a certain genomic distance cut-off, defined by the user [9]. Methods for directly profiling transcription factor binding can provide more direct evidence of regulatory behaviour, but are currently limited to assaying only a few transcription factors simultaneously within the same cell [70–75].

Incorporating temporal information into multiomics assays may help to resolve the relationship between *cis*-regulation and gene expression. Changes to the accessibility of *cis*-regulatory elements should precede changes to the expression of regulated genes, and this relationship has been developed into the concept of ‘chromatin potential’ [76]; integrating metabolic labelling into multiomics assays may further provide a ‘dynamical’ bridge between upstream chromatin regulation and resulting gene expression consequences.

Similarly, chromatin information may help to better resolve the inference of gene expression dynamics.

Cell states can be distinguished based on differential gene expression or by differential chromatin accessibility, and these two may not exactly align [77]. Two cell states with different expression profiles may have similar chromatin landscapes, or they may substantially differ, and the regulatory dynamics of these two scenarios may differ drastically. As such, information on a cell’s chromatin landscape may be required to properly distinguish dynamics that may not be resolvable from an RNA-only perspective.

This approach may offer a valuable use of chromatin accessibility data that is less subject to the difficulties of predicting specific transcription factor binding patterns from the openness of chromatin: ascertaining whether two cells share the same overall chromatin landscape can indicate whether we can expect their gene regulatory networks to be wired similarly (and thus, these cells can be modelled together) or not (in which case, the two cells may have different functional gene regulatory networks and different energy landscapes).

Even with the more complete picture provided with time-resolved or multi-modal data, a proper understanding of causal relationships in gene regulation will require interventionalist studies. For this, methods for high-throughput genetic perturbation combined with single-cell sequencing, such as Perturb-seq [78], are commonly used to assay the complex network of interactions in biological systems.

In such studies, a dynamical perspective would be particularly valuable: by introducing perturbations at specific times, and by using time-resolved sequencing (this latter development recently done with PerturbSci–Kinetics, which combines perturb-seq with metabolic labelling [79]), one could better capture the cascading ‘butterfly effect’ of consequences from a perturbation, and distinguish direct interactions from correlative effects. Such an approach could also better handle the time-dependent nature of genetic interactions, determining not just whether two genes interact but also the time and context in which this interaction takes place.

Of course, including an additional axis of variation—not just what genes are perturbed, but *when* this perturbation occurs—adds layers of complexity to experimental set-ups, provides new challenges for interpreting data, and more thinly spreads the throughput of single-cell assays across many more conditions. Moreover, such endeavours will require perturbational methods that can alter cellular behaviour with precision and temporal resolution.

Methods such as sci-plex [80] and MULTI-seq [81] can be used to multiplex hundreds of conditions into a single sequencing run, providing feasible experimental set-ups for dynamical perturbation experiments. Moreover, technologies for perturbation of gene expression and protein activity continue to offer increasing levels of control in the interventions that can be done: for example, inducible CRISPR screens [82], libraries of degron tags for destabilization and proteolysis [83], and, more recently, these two technologies combined for analogue modulation of gene expression [84]. More generally, optogenetic tools can provide perturbations that are rapid, reversible, and precisely controllable in both time and space [85].

Indeed, the behaviour of gene regulation in space is another area that will be important to consider, and the rapid development of spatially-resolved transcriptomics technologies presents opportunities to explore the spatial constraints of gene regulation [86,87]. However, the addition of spatial information brings considerable challenges as well. Two-dimensional sections captured in these approaches need to be stacked in a third dimension and subsequently stacked through time with a sufficiently dense time-series; these timepoints will come from different tissues or organisms, introducing an additional source of confounding noise in the form of inter-organismal variation in tissue size, developmental timing or disease state. Then, cellular ‘movement’ through gene expression space will need to be integrated with physical cellular movement in a parsimonious and biophysically meaningful manner that can handle the possibility of transcriptomic state affecting spatial location and vice versa.

Connecting the dynamical analysis of gene expression trajectories to their spatial context *in vivo* presents experimental, computational and theoretical challenges, but can provide material benefit to the accuracy of gene regulation modelling. Spatial information can serve to constrain the picture of expression dynamics, helping to distinguish possible from impossible dynamics in systems that would be otherwise underdetermined from single-cell data alone. Work in this direction is ongoing: for example, TEMPOmap [88] provides a method for parallel metabolic labelling and spatial transcriptomics, while Spatio [89] provides tools to model the ‘morphometric’ vector field of time-coursed spatial data. In parallel, the continued improvement of organoid [90,91] and *in vitro* embryo [92] models may provide three-dimensional structured tissues that are far more experimentally tractable for the level of high-throughput data collection that would be required for spatial dynamical modelling. An additional strength of these *in vitro* systems is that they allow greater control and knowledge of the signalling environments that cells in an experiment are exposed to, allowing a more explicit relationship to be mapped between input signals and output expression dynamics.

**Figure 2.**
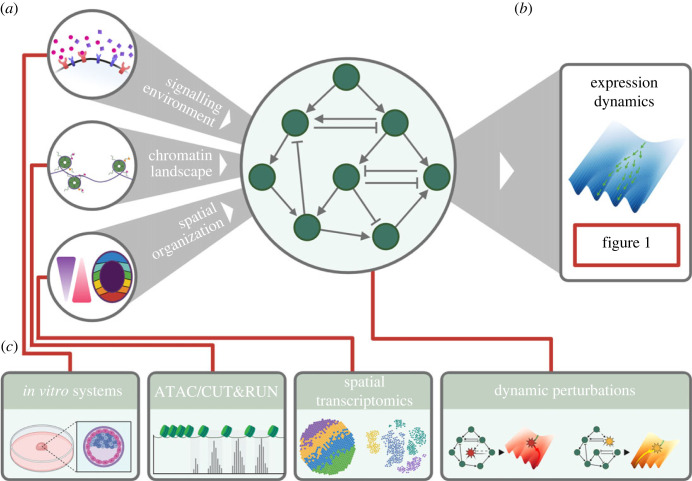
Inputs, outputs and experimental methods for gene regulatory networks. (*a*) The behaviour and function of gene regulatory networks are controlled by various inputs and constraints, including: (i) the signalling environment of the cell that serves as input to transcription factor networks, (ii) the epigenetic landscape that determines how transcription factors can operate, and (iii) the spatial organization of interacting cells in a tissue. (*b*) These inputs and constraints can be thought to determine the structure of the regulatory network’s energy landscape, which dictates the dynamics of its behaviour, measurable through time resolved RNA sequencing. (*c*) Experimental methods for measuring and manipulating gene regulatory networks: with *in vitro* systems, signalling environments can be carefully controlled; epigenetic profiling methods such as ATAC and CUT&Run can provide information on transcription factor binding and activity; spatial transcriptomics can capture patterning and organization, while high throughput perturbation methods can alter the structure and thus function of regulatory networks.

Modern genomics technologies have led to a huge increase in the sensitivity and throughput of our descriptions of cellular and genetic dynamics (summarized in figure 2). We can measure both molecular and cellular measurements of gene expression dynamics, and can connect this gene expression to epigenetic, perturbational and spatial contexts, providing a picture of gene regulation dynamics that is detailed beyond what would have seemed possible only a couple decades ago. Work to integrate all these modalities into a single assay continues to grow our capabilities, for example with the recently published DARLIN mouse model [93] which allows profiling of RNA, chromatin accessibility, lineage information and DNA methylation from single cells isolated from *in vivo* mouse tissue. Such technologies will be vital to grapple the full extent of biological systems. However, rather than providing clarity, these highly-resolved descriptions can often serve to emphasize just how complex the systems we hope to understand really are. Indeed, while our ability to describe the complexity of cellular systems has improved, we have not seen a comparable transformation in the tools we use to distil this complexity into intuition of how these systems actually work. As such, a final question remains: how can we construct an intuitive, predictive understanding of complex systems of gene regulation?

## 6. Causal challenges

To understand the causal relationships that connect regulatory genes to cellular behaviour, it is necessary to resolve an adequate picture of the cell’s gene expression dynamics. What is less clear is whether this is *sufficient*. While the decision making of cells can be relatively simple—for example, a hierarchy of binary fate decisions in development, or a binary presence or absence of a particular phenotype—the gene regulatory networks that control them can be much more complex.

We can take as an example the developing mammalian neural tube, where 11 different domains of progenitor cells form along the dorsoventral axis in response to morphogen signals [94]. These domains can be distinguished through a ‘code’ of dozens of transcription factors with highly overlapping expression patterns [2]. On top of this, when these progenitor cells start to form neurons, a ‘temporal code’ of transcription factors distinguishes the neurons formed in each domain based on the day of their formation [95]. The gene regulatory networks that control neuronal formation in the neural tube may thus involve hundreds of interactions between several dozens of transcriptions factors.

Meanwhile, mathematical models of even a handful of genes in a regulatory network encounter issues of structural non-identifiability (where it is not possible to find a unique solution based on the output and structure of the model equations [96,97]) and model ‘sloppiness’ (where parameters can change radically without changing model behaviour [98]). Not only can different models produce the same behaviour, but qualitatively different behaviours can be produced by a single model structure [99], suggesting that even if the structure of a network is known, we still may not be able to fully understand its function or behaviour. These issues become increasingly severe as the complexity of the model grows and, importantly, are not necessarily consequences of the data, but of the structure of the models themselves.

As such, it is reasonable to ask whether seeking to model the complete, exact structure of gene regulatory networks is a feasible goal. Large network models trained on single-cell and multiomics data can provide useful insights, but these models fall short of providing a truly predictive, intuitive understanding of the system. This is not a fault of these models *per se*, but an issue of feasibility of their goal.

It may thus be necessary to abstract some of the complexity of biological systems, learning high-level representations of regulatory networks and modelling causal relationships within these representations. This approach could be considered analogous to coarse grained modelling, an approach used extensively for molecular modelling in physics and chemistry [100,101]. By using a principled abstraction of molecular behaviours, coarse-grained models can provide a useful, predictive descriptions without having to handle the entire complexity of the system explicitly.

Importantly, higher-level abstractions might not just be easier to learn, they might be more informative, too [102]. In complex systems, function does not have to arise solely from the action of individual components, it can manifest as an emergent property of the system’s structure itself.

For example, one could consider a digital image of a face. In this example, we can see that no individual pixel in the image contains sufficient information to explain that there is a face in the image—at this level of detail, there is no direct causal relationship between individual pixels and the presence of a face. The contents of the image only emerge from higher-level relationships between collections of pixels. To understand the image, we must consider more abstract structures: ‘eye’ pixel-groups, ‘nose’ pixel-groups and so on.

Genes to cellular systems may be as pixels to images; the classical, reductionist view of genes as units of causality in biological systems may be insufficient to provide satisfactory descriptions of how global gene regulation works. Moreover, key properties of causality—such as stability, proportionality, specificity—may only emerge when we consider the relationships at this higher level, rather that between individual genes [14].

Such a view of gene regulation provides new considerations: for example, it is important not just to consider the components of a system, but also the constraints [14,103]. These can be any factors that restrict the system’s degrees of freedom and ‘canalise’ a particular behaviour in a particular context. Chromatin landscape, as discussed above, could be seen as a constraint for how a gene regulatory network is wired. Other constraints could include spatial organization, biophysical properties, or signalling environments. Properly defining a system’s inputs, constraints and outputs may be necessary to determine the right level of coarse-grained abstractions to use.

Capturing the dynamics of the system will still be necessary for this higher-level view of gene regulation. It will still be vital to track the complex behaviour of these large, nonlinear, feedback-driven systems in order to learn simplified or abstract representations. Indeed, emergent behaviours of gene regulatory networks have been found and studied in systems biology: the toggle switch [104], the repressilator [105] and the AC/DC circuit [106] are all examples of network topologies with specific functions and outputs that are not interpretable from individual components alone, and all have been proposed to have functions in biological systems. Critically, these motifs are all *dynamical concepts*, and cannot be properly understood without a highly resolved picture of their temporal behaviours. Ultimately, causal properties of gene regulatory networks can be thought of as dynamical regimes that connect the regulatory network to the biological outcome [14].

**Figure 3.**
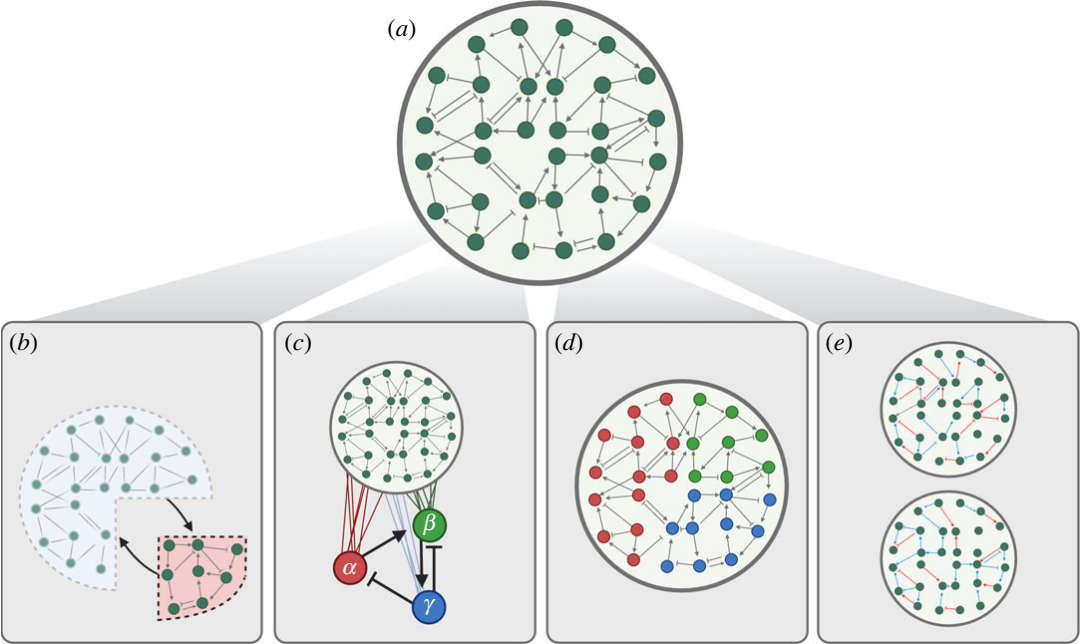
Learning abstract representations of complex gene regulatory networks. (*a*) The full extent of regulatory systems can be complex, with very many interacting parts. Explicitly modelling every component of these systems may not be feasible, and methods to abstract some of this complexity away may be of value for studying biological function. Some examples of possible approaches include the following. (*b*) Separating networks into relevant and bulk sub-networks that interact, for example through Mori–Zwanzig formalism [107]. (*c*) Causal representation learning [108], where a latent representation of the system is learnt, and causal structure within this representation, rather than the entire system, is inferred. For example, latent factor causal models [109] model gene regulatory networks through the interaction of unobserved latent factors that cluster genes. (*d*) The components of a gene regulatory network can be split into modules or gene programmes which can then themselves be studied, for example [110] and [111]. (*e*) Focussing not on structure but on change: for example, Difference causal inference [112] models the changes in a gene regulatory network's interactions between two conditions rather than the entire explicit structure.

This approach to modelling gene regulation will certainly be challenging, but there is already a wealth of research that can guide the way (some examples given in figure 3). Inspiration can be taken from concepts in statistical physics and chemistry, such as coarse-grained modelling [100,101], Mori–Zwanzig methods for dividing systems into relevant and irrelevant components [107], or rule-based languages for constructing mechanistic models of molecular dynamics [113]. Opportunities may also come from the integration of single-cell genomics and generative machine learning methods. In particular, emerging research in causal representation learning [108,114] explores the application of methods already common in single-cell research, such as variational autoencoders, to the problem of learning high-level representations of low-level data, alongside structural causal models of these representations.

Single-cell analysis is already moving towards interpretability and higher-level representations, with work on inferring ‘gene modules’ for RNA velocity analysis [50]; ‘gene programmes’ for cell type analysis [110] or reference mapping [111]; and ‘meta-cells’ for clustering [115]. These works highlight the directions in which analyses will need to move, but further developments are required to move from interpretable representations derived from user-defined lists or genetic correlations to methods specifically designed to address causality in the context of the regulatory control of cell behaviour; from context-agnostic representations to representations that are specifically relevant to the biological question at hand.

With rapidly growing capabilities for genome-wide, high-throughput and multi-modal assays, it is increasingly possible to collect thousands of measurements across millions of data points; however, our own understanding of biology cannot be thousand-dimensional, and so new tools and methods are required to convert these high-dimensional observations into simple, intuitive explanations.

## 7. Conclusion

In 2017, Jonas and Kording asked: could a neuroscientist understand a microprocessor? [116]. In this study, they applied conventional experimental methods and analyses of neuroscience to study a microprocessor (a system with a complex but perfect ground truth) and found that from these techniques, they could not provide a satisfying explanation of how the microprocessor actually worked. Thus, they concluded, new ways of tackling the problem were needed if neuroscience was to understand the brain.

It could be said that single-cell analysis of gene regulation is in a similar situation: we have gathered incredible insight into complex phenotypes in development and disease, but in many cases, we still lack a satisfying, mechanistic understanding of the underlying regulatory networks. A more dynamical perspective will be an important step towards this understanding, providing insight into the dynamic regimes of gene expression that connect genotype to phenotype, and helping to resolve causal relationships in the data. Connecting these dynamical recordings to data modalities describing spatial, epigenetic and signalling contexts can inform our analyses about the factors that constrain a system’s dynamics, while gene manipulation technologies provide the means to partner high-throughput measurements with high-throughput perturbations. Yet even with these developments, it may not be feasible to model entire regulatory networks explicitly, and there is no guarantee that such models would provide an satisfying causal description of biological function. As we employ a dynamical perspective of biology to better capture causal relationships, it may be that we need to update our understanding of causality to handle the complex dynamics of biology.

## Data Availability

This article has no additional data.
